# Association between incidence of dental caries and body mass index among preschool children: a longitudinal study

**DOI:** 10.1590/1807-3107bor-2026.vol40.042

**Published:** 2026-07-24

**Authors:** Maria Eliza da Consolação Soares, André Souza Rufino, Júlia Fernandes Siqueira, Valéria Silveira Coelho, Thiago Motta Rego, Maria Letícia Ramos Jorge, Joana Ramos Jorge

**Affiliations:** (a) Universidade Federal de Juiz de Fora – UFJF, School of Dentistry, Department of Pediatric Dentistry, Governador Valadares, MG, Brazil.; (b) Universidade Federal de Minas Gerais – UFMG, School of Dentistry, Department of Pediatric Dentistry, Belo Horizonte, MG, Brazil.; (c) Universidade Federal dos Vales do Jequitinhonha e Mucuri – UFVJM, Department of Pediatric Dentistry, School of Dentistry, Diamantina, MG, Brazil.

**Keywords:** Body Mass Index, Dental Caries, Child, Preschool, Longitudinal Studies

## Abstract

Body mass index (BMI) disorders and dental caries are common and interrelated conditions in children, sharing risk factors such as inadequate diet and socioeconomic factors. This longitudinal study was designed to improve understanding of the determinants of children's health and, consequently, to promote health from childhood. The research was conducted between August 2017 and November 2018 in Diamantina, Brazil, and investigated the association between the incidence of dental caries and BMI in preschool children. The final sample included 122 children aged three to four years and 11 months, who were evaluated at two points over a one-year interval. The analysis showed that reduced family income (RR = 3.94; 95%CI: 1.26–12.39) and the incidence of caries (RR = 1.86; 95%CI: 1.01–3.42) were significant risk factors for low weight. Although maternal education initially appeared relevant (RR = 4.67; 95%CI: 1.48–14.73), its influence diminished when family income was considered. In conclusion, this study found an association between incidence of dental caries and low BMI (underweight) in preschoolers after one year of follow-up. The incidence of caries and the reduction or maintenance of low monthly family income were identified as risk factors for low weight in preschoolers during one year of follow-up.

## Introduction

Obesity and underweight, often assessed using Body Mass Index (BMI), are public health issues that pose risks to human health.^1^ These conditions have a multifactorial and complex etiology,^2^ similar to dental caries. Dental caries is the most prevalent oral disease worldwide, affecting an estimated 621 million children.^3^ A recent systematic analysis for the Global Burden of Disease Study 2021 showed that global trends in the prevalence of untreated caries in deciduous teeth remained unchanged between 1990 and 2021.^4^ A diet rich in fermentable carbohydrates, processed foods, and low nutritional value is a common risk factor associated with BMI variations and dental caries in childhood.^5,6^ Consequently, studies have investigated whether there is an association between BMI variations and dental caries, but the findings remain conflicting.

Some studies have identified a potential association between dental caries and obesity.^7,8^ This link is often attributed to high sucrose intake, which can contribute to both obesity and dental caries.^9,10^ Conversely, other studies have reported an inverse relationship, with higher BMI associated with a lower prevalence of caries.^11-13^ Additional research has found a connection between dental caries and low BMI.^14,15^ Socioeconomic status may serve as a common risk factor linking these conditions.^16,17^ However, it is also possible that dental caries precede the onset of low weight, as the association between these conditions might be explained by masticatory difficulties resulting from severe caries lesions.^18,19^ Some studies have found no association between these conditions.^20-22^

Systematic reviews^23,24^ have not reached a consensus on the associations between dental caries and BMI in children. However, Chen et al.^24^ identified an association between dental caries and obesity in high-income countries. Investigating this topic is crucial, as the findings can help professionals address health issues in an interdisciplinary manner. Furthermore, dental caries affects almost 47.0% of Brazilian children aged five years,^26^ and childhood obesity has increased in prevalence in recent years in that country.^27^ Thus, studying this topic with more robust study designs is also significant for developing public health strategies to improve the population's quality of life. This study aims to evaluate the association between the incidence of dental caries and changes in BMI among preschool children over one year of follow-up. Additionally, it assessed the influence of socioeconomic factors as potential confounders.

## Methods

The preparation of this study followed the STROBE guidelines for cohort studies. This study is part of a larger project aimed at evaluating the masticatory performance of preschool children before and after dental treatment. The project was approved by the Human Research Ethics Committee of the Federal University of Vales do Jequitinhonha e Mucuri, Brazil (Protocol number CAAE 830023018.0.0000.5108). Data were collected from August 2017 to November 2018. All guardians of the participating children were fully informed about the study's objectives and signed a written informed consent form.

### Sample and study design

Data collection took place at the Pediatric Dentistry clinics of the Federal University of Vales do Jequitinhonha e Mucuri (UFVJM) with children aged 3 to 4 years and 11 months. The participants were selected from public and private preschools and daycare centers in Diamantina, MG, Brazil.

This project was a prospective longitudinal study. The study population included children aged 3 to 4 years and 11 months at baseline, of both sexes, enrolled in public and private daycare centers and preschools in Diamantina, Minas Gerais. At the end of the observation period, the population was divided into two groups: one consisting of children who developed dental caries and the other of those who did not. The allocation of children into each group was determined through prior examination and assessment. This assessment, which evaluated the presence of dental caries, was conducted at daycare centers and preschools in the city after obtaining parental consent.

This study was part of a broader investigation aimed at evaluating masticatory performance before and after dental caries treatment in preschool children. Considering the variables involved, a sample size calculation was conducted, and the statistical power for the previously defined sample size was determined.

Masticatory performance parameters before and after dental caries treatment were obtained from a pilot study involving 20 children: 10 children with cavitated carious lesions were matched by sex and age with 10 children without cavitated lesions. The masticatory performance of the group without carious lesions was assessed during the same period as their matched pairs with carious lesions. Using a standard deviation of 0.32 - corresponding to the mean standard deviation of the median particle size across group evaluations - a detectable difference of 0.2, a statistical power of 90%, and a standard error of 5%, the minimum required sample size was calculated as 54 children per group. To account for potential losses, 12 additional children were included in each group, resulting in a total study population of 136 children. The sample size calculation was performed using the OpenEpi platform, version 3.01^19^ (Figure). Only 12 children were included to minimize the occurrence of losses because these children were selected from a database of a previous study^28^ with 285 children, of whom 136 met the eligibility criteria for the present investigation.

**Figure 1:**
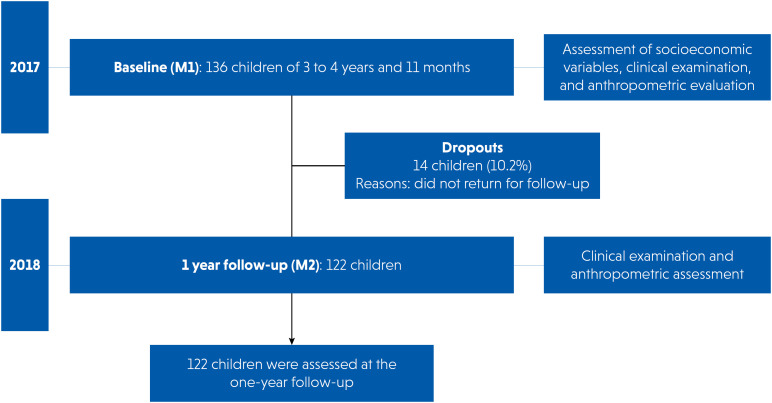
Flowchart of the study

The study included children in the primary dentition phase, aged 3 to 4 years and 11 months, regardless of sex, enrolled in public and private daycare centers and preschools in Diamantina, MG. Exclusion criteria applied to children who: had systemic conditions (e.g., syndromes or neurological disorders) as reported by parents/caregivers; had erupting first molars; were using orthodontic appliances; or showed any signs or symptoms of pulp involvement, such as spontaneous pain during the initial examination, abscesses, or fistulas.

A trained team conducted the research, consisting of a dentist who performed all oral clinical examinations and an assistant. The team underwent a training and calibration process that included both theoretical and practical components. This process involved training on dental caries and malocclusion examinations, including review of diagnostic criteria and analysis of photographs. Fifteen children were first examined by an experienced professional and then by the dentist responsible for the study to establish inter-examiner calibration (Kappa = 0.77 for dental caries and 0.89 for malocclusion). One week later, the same dentist re-examined the same group of children to evaluate intra-examiner reliability (Kappa = 0.81 for dental caries and 0.92 for malocclusion). The children involved in this training were not included in the main study.

### Data collection

All assessment instruments were applied at two time points for the study sample. Evaluations through clinical examination and anthropometric measurements were carried out both at the beginning of the follow-up period (M1) and at the end (M2). Additionally, two forms were used for data collection: one to record the oral clinical examination and another to document sociodemographic and economic factors, such as maternal education and household income.

### Clinical examination

Clinical examinations were performed under artificial light from a reflector, using personal protective equipment (PPE), in accordance with the biosafety standards in place at the time of data collection. The procedures used a clinical mirror, a millimeter-marked periodontal probe, and a WHO probe. The clinical examination was conducted after the examiner brushed the teeth with fluoride toothpaste, followed by drying the teeth with sterile gauze.

Dental caries was assessed using the International Caries Detection and Assessment System (ICDAS).^29^ The ICDAS is a tool designed to identify caries lesions and their severity, classifying lesions based on the progression of cavitation.^29^ Using this system, the number of teeth affected by caries in each child in the sample was determined, specifying the extent of each lesion. Both cavitated and non-cavitated caries lesions, as well as active and inactive lesions, were considered to determine the incidence of caries.

The classification for occlusal problems in primary dentition was defined according to the criteria proposed by Foster and Hamilton in 1969.^30^ In this study, anterior open bite, posterior crossbite, anterior crossbite, and excessive overjet were evaluated. All assessments were conducted with the teeth in occlusion.

### Anthropometric assessment

Each child's weight was measured using a G-Tech Glass G4FB digital scale (Accumed Produtos Médico Hospitalares Ltda, Rio de Janeiro, Brazil), calibrated to an accuracy of 100 grams (g). A deduction of 200 g was made from each child's weight to account for clothing. Height was measured using a portable stadiometer with a vertical adjustable rod (WCS, Cardiomed, Curitiba, Brazil). Children were positioned with their backs against the stadiometer, ensuring the Frankfurt Plane was parallel to the ground. The movable part of the stadiometer was placed at the highest point of the head. For both weight and height assessments, children were barefoot, with their feet together and shoulders straight.

BMI was calculated using the recommended formula, dividing weight (kg) by height (m) squared (BMI = weight/height^2^). The resulting BMI values were plotted on a WHO-recommended growth curve, which accounts for the child's age and sex. This process determined the Z-scores for each child. Children with Z-scores above +1 are considered at risk of overweight; those with Z-scores above +2 and +3 are classified as overweight and obese, respectively. Children with Z-scores below -2 are considered underweight, and those with Z-scores below -3 are considered malnourished. Children with Z-scores from -2 to +1 are considered to have normal weight.^31^

### Statistical analysis

Data analysis was performed using the Statistical Package for the Social Sciences (SPSS), version 24.0, and included descriptions of variable frequencies at baseline and follow-up. Unadjusted and adjusted Poisson regression with robust variance was used to assess the association between caries incidence and BMI changes, as well as to control for confounding factors. The unadjusted analysis was used to exclude variables with a p-value ≥ 0.20. However, variables with a p-value ≥ 0.20 that were considered by the team to be important confounding factors for the outcome were included. Only explanatory variables with a p-value < 0.05 after adjustment were selected for the final model. Relative risk (RR) and 95% confidence intervals (95%CI) were calculated.

## Results

The final sample consisted of 122 children who had a normal BMI or were underweight at follow-up (89.70%) (Figure). Fourteen children did not return for follow-up. Table 1 presents the descriptive analysis of the variables at baseline and/or follow-up according to BMI at follow-up.

**Table 1:** Descriptive analysis of variables at baseline and/or follow-up according to BMI at follow-up.

Covariates		BMI at follow-up		
		Normal (%)	Underweight (%)	Overweight obesity (%)
Age (baseline) (months)				
	≥ 48	48 (49.0)	14 (58.3)	13 (92.8)
	> 48	50 (51.0)	10 (41.7)	1 (7.2)
Sex				
	Female	48 (49.0)	12 (50.0)	6 (42.8)
	Male	50 (51.0)	12 (50.0)	8 (57.2)
Maternal education (baseline)				
	Higher education	30 (30.6)	3 (12.5)	1 (7.2)
	High school	49 (50.0)	7 (29.2)	9 (64.3)
	Elementary school	19 (19.4)	14 (58.3)	4 (28.5)
Monthly family income (baseline and follow-up)				
	Stayed high	40 (40.8)	4 (16.7)	3 (21.5)
	Increased	22 (22.4)	1 (4.2)	3 (21.5)
	Decreased	8 (8.2)	5 (20.8)	4 (28.5)
	Stayed low	28 (28.6)	14 (58.3)	4 (28.5)
BMI (baseline)				
	Normal	49 (50.0)	10 (41.7)	8 (57.2)
	Overweight	24 (24.5)	12 (50.0)	5 (35.6)
	Underweight	25 (25.5)	2 (8.3)	1 (7.2)
Dental caries incidence (baseline and follow-up)				
	No	85 (86.7)	16 (66.7)	13 (92.8)
	Yes	13 (13.3)	8 (33.3)	1 (7.2)

Table 2 shows both unadjusted and adjusted analyses of the factors associated with underweight BMI at follow-up. The unadjusted analysis revealed that lower maternal education (RR = 4.67; 95%CI: 1.48–14.73), a reduction in monthly household income (RR = 4.23; 95%CI: 1.33–13.50), low income at both baseline and follow-up (RR = 3.67; 95%CI: 1.31–10.25), and the incidence of dental caries (RR = 2.40; 95%CI: 1.19–4.87) were risk factors for underweight at follow-up (Table 2).

**Table 2:** Unadjusted and adjusted analysis of factors associated with underweight BMI at follow-up.

Covariates		n (%)	Unadjusted analysis		Adjusted analysis	
			RR (95%CI)	p-value	RR (95%CI)	p-value
Age (baseline)						
Mean (SD)		47.95 (7.9)	0.99 (0.95–1.04)	0.852		
Sex						
	Female	60 (49.2)	1.00			
	Male	62 (50.8)	0.97 (0.47–1.98)	0.929		
Maternal education (baseline)						
	Higher education	33 (27.0)	1.00			
	High school	56 (45.9)	1.37 (0.38–4.96)	0.626		
	Elementary school	33 (27.0)	4.67 (1.48–14.73)	0.009		
Monthly family income (baseline and follow-up)						
	Stayed high	44 (36.1)	1.00		1.00	
	Increased	23 (18.9)	0.48 (0.06–4.03)	0.498	0.50 (0.06–4.26)	0.524
	Decreased	13 (10.7)	4.23 (1.33–13.50)	0.015	3.94 (1.26–12.39)	0.019
	Stayed low	42 (34.4)	3.67 (1.31–10.25)	0.013	3.40 (1.22–9.45)	0.019
BMI (baseline)						
	Normal	59 (48.4)	1.00			
	Overweight	36 (29.5)	1.97 (0.95–4.08)	0.069		
	Underweight	27 (22.1)	0.44 (0.10–1.86)	0.263		
Dental caries incidence (baseline and follow-up)						
	No	101 (82.8)	1.00		1.00	
	Yes	21 (17.2)	2.40 (1.19–4.87)	0.015	1.86 (1.01–3.42)	0.046

In the final Poisson regression model, a reduction in monthly household income (RR = 3.94; 95%CI: 1.25–12.38), low income at both baseline and follow-up (RR = 3.40; 95%CI: 1.22–9.44), and the incidence of dental caries (RR = 1.86; 95%CI: 1.01–3.42) remained risk factors for underweight at follow-up.

This analysis was only possible by considering the change in BMI to the underweight category, since only one child presented with both dental caries and overweight/obesity during the follow-up period, which limited analyses involving higher BMI categories.

## Discussion

This study demonstrated that the risk factors for low BMI (underweight) at follow-up were a reduction in family income, persistent low income, and the occurrence of dental caries. These results highlight the relationship between dental caries and BMI, which is often attributed to shared risk factors. However, the findings of this study suggest a different perspective for explaining this association.

A reduction in family income is a determining factor that can significantly affect children's nutritional health, leading to underweight. This is due to several reasons, including food insecurity.^32^ Studies indicate that low income increases the prevalence of food insecurity within families, as households with reduced income face difficulties in purchasing sufficient and nutritious food, resulting in inadequate intake of calories and essential nutrients needed for children's healthy growth and development.^32,33^ Moreover, low-income families often consume ultraprocessed foods that are rich in sugars and fats but low in micronutrients essential for healthy growth.^34^ Another important factor is the cost of foods necessary for a balanced diet, which often exceeds the financial capacity of families with reduced income. The cost of basic food in various Brazilian capitals consumes, on average, 54.0% of the salary of a worker earning the minimum wage.^35^ Due to this financial imbalance, families are forced to buy cheaper foods that are less nutritious, increasing the risk of underweight and other health problems associated with malnutrition.^36^

Although maternal education is a crucial factor for knowledge and health practices, this variable lost its significance when associated with low income in the same statistical model. Gundersen and Ziliak^33^ demonstrate that when controlling for income, the significance of maternal education in statistical analyses of child health often decreases, suggesting that income is a more immediate and direct determinant of living conditions that affect children's health. This occurs because there is a direct proportional relationship between education and income. In other words, individuals with higher education levels tend to secure better job opportunities and, consequently, higher income.^38^ Thus, since these variables were included together in the model of the present investigation, family income was more important for the outcome analyzed.

The lack of association between a child's weight at baseline and after one year suggests that children's weight can vary significantly over short periods, reflecting the dynamic nature of growth during childhood. In the early years of life, children can rapidly gain weight and height, leading to significant BMI changes in brief periods.^1^ Additionally, studies show that lifestyle interventions, such as changes in socioeconomic conditions, the home environment, and participation in physical activity, can result in significant BMI changes within a few months, highlighting the influence of these factors on children's growth.^38,39^

Studies have shown an association between the incidence of dental caries and both high and low BMI in children.^9,13^ Acs et al.^40^ and Ayhan et al.^41^ found that children with severe caries had significantly lower weight than children without caries. This result was also observed in the present study, as the incidence of caries remained a risk factor for low weight in children. Research indicates that children with dental caries consume fewer fruits and vegetables and more processed and sugary foods, negatively impacting their nutritional status, as these foods are poor in essential nutrients and can lead to malnutrition and low weight.^42,43^ Moreover, children with cavitated caries lesions may reduce their eating frequency due to pain, resulting in weight loss. A study conducted by Losso et al.^44^ demonstrated that pain and infection associated with caries can alter eating habits, affecting the growth pattern of children, and influencing low BMI.

Considering the common causal relationship between dental caries and overweight or obesity, cross-sectional studies may identify an association between these conditions. However, in longitudinal studies, it is reasonable to assume that children with dental caries, especially when the dentin is exposed, may reduce their sugar intake due to pain caused by exposed microscopic tubules. Consequently, these children may experience weight loss over time. Furthermore, diets predominantly rich in fatty foods, which are also considered obesogenic, are not direct risk factors for the development of dental caries. This may partially explain the heterogeneity observed in the association between obesity and caries among the studies.

Efforts were made to contact all the children who participated in the baseline study; 14 could not be found. Three children moved to another city and 11 changed their phone numbers and addresses, making it impossible to locate them. All of these children had normal BMI, and only one had dental caries at baseline. Thus, it is possible that the data from these children do not have a relevant impact on the results.

This study has some limitations that should be considered when interpreting the results. The sample size was relatively small, and the lack of representativeness may limit the generalization of the findings to the broader population. Additionally, the outcome analyzed focused exclusively on low weight, without considering other possible nutritional or health variables that could provide a more comprehensive view of the child's nutritional status. Another important limitation is the absence of information regarding the children's physical activity levels, which may act as a confounding factor, since physical activity directly influences energy balance, growth patterns, and nutritional status. The short follow-up period of just one year may not be sufficient to capture all the variations and trends in the development and nutritional status of these children. Therefore, future studies with larger sample sizes, multiple outcomes, and longer follow-up periods are recommended to validate and expand the conclusions presented here.

Despite its limitations, this study broadens understanding of the relationship between BMI and dental caries in childhood and reinforces the importance of developing integrated preventive strategies, with an emphasis on promoting healthy eating habits.

## Conclusion

This study found an association between the incidence of dental caries and low BMI (underweight) in preschoolers after one year of follow-up. The incidence of caries and a reduction or maintenance of low monthly family income were identified as risk factors for low weight in preschoolers during one year of follow-up. The child's weight at baseline and follow-up was not associated with low weight. Further studies should be conducted with a larger sample size, a longer follow-up period, and multiple outcomes to more accurately assess this association.

## Data Availability

The authors declare that all data generated or analyzed during this study are included in this published article.

## References

[1] World Health Organization (2000). Obesity: preventing and managing the global epidemic: report of a WHO consultation. World Health Organization.

[2] Saporiti JM, Vera BS, Arruda BS, Caldeira VD, Pereira LG, Nascimento GG (2014). Obesidade e saúde bucal: impacto da obesidade sobre condições bucais. Rev Fac Odontol (Univ Passo Fundo).

[3] Kassebaum NJ, Bernabé E, Dahiya M, Bhandari B, Murray CJ, Marcenes W (2015). Global burden of untreated caries: a systematic review and metaregression. J Dent Res.

[4] Bernabé E, Marcenes W, Suliankatchi Abdulkader R, Guimarães Abreu L, Afzal S, Alhalaiqa FN (2025). Trends in the global, regional, and national burden of oral conditions from 1990 to 2021: a systematic analysis for the Global Burden of Disease Study 2021. Lancet.

[5] Ribeiro CC, Silva MC, Nunes AM, Thomaz EB, Carmo CD, Ribeiro MR (2017). Overweight, obese, underweight, and frequency of sugar consumption as risk indicators for early childhood caries in Brazilian preschool children. Int J Paediatr Dent.

[6] Kennedy T, Rodd C, Daymont C, Grant CG, Mittermuller BA, Pierce A (2020). The association of body mass index and severe early childhood caries in young children in Winnipeg, Manitoba: a cross-sectional study. Int J Paediatr Dent.

[7] Manohar N, Hayen A, Fahey P, Arora A (2020). Obesity and dental caries in early childhood: a systematic review and meta-analyses. Obes Rev.

[8] Piovesan ÉT, Leal SC, Bernabé E (2022). The relationship between obesity and childhood dental caries in the United States. Int J Environ Res Public Health.

[9] Davidson K, Schroth RJ, Levi JA, Yaffe AB, Mittermuller BA, Sellers EA (2016). Higher body mass index associated with severe early childhood caries. BMC Pediatr.

[10] Elger W, Kiess W, Körner A, Schrock A, Vogel M, Hirsch C (2019). Influence of overweight/obesity, socioeconomic status, and oral hygiene on caries in primary dentition. J Investig Clin Dent.

[11] Hooley M, Skouteris H, Boganin C, Satur J, Kilpatrick N (2012). Body mass index and dental caries in children and adolescents: a systematic review of literature published 2004 to 2011. Syst Rev.

[12] González Muñoz M, Adobes Martín M, González de Dios J (2013). Revisión sistemática sobre la caries en niños y adolescentes con obesidad y/o sobrepeso. Nutr Hosp.

[13] Rodríguez G, Cabello R, Urzúa I, Reyes M, Faleiros S, Ruiz B (2017). Asociación entre lesiones de caries y estado nutricional en niños preescolares de Santiago, Chile. Int J Odontostomatol.

[14] Shi R, Lin C, Li S, Deng L, Lin Z, Xiu L (2022). Obesity is negatively associated with dental caries among children and adolescents in Huizhou: a cross-sectional study. BMC Oral Health.

[15] Hao C, Hao Y, Lou X, Wang X, Liu W, Zhou H (2024). Secular trends of dental caries and association with nutritional status: a retrospective analysis of 16,199 Chinese students from three successive national surveys from 2010 to 2019. Front Public Health.

[16] Shen A, Bernabé E, Sabbah W (2020). Severe dental caries is associated with incidence of thinness and overweight among preschool Chinese children. Acta Odontol Scand.

[17] Large J, Marshman Z (2022). Does dental caries lead to stunting and wasting in children?. Evid Based Dent.

[18] Sheiham A (2006). Dental caries affects body weight, growth and quality of life in pre-school children. Br Dent J.

[19] Soares ME, Ramos-Jorge ML, de Alencar BM, Oliveira SG, Pereira LJ, Ramos-Jorge J (2017). Influence of masticatory function, dental caries and socioeconomic status on the body mass index of preschool children. Arch Oral Biol.

[20] Kumar S, Kroon J, Lalloo R, Kulkarni S, Johnson NW (2017). Relationship between body mass index and dental caries in children, and the influence of socio-economic status. Int Dent J.

[21] Paisi M, Kay E, Kaimi I, Witton R, Nelder R, Potterton R (2018). Obesity and caries in four-to-six year old English children: a cross-sectional study. BMC Public Health.

[22] Fernandes TO, Carvalho PA, Abreu FV, Kirschneck C, Küchler EC, Antunes LS (2023). Association between nutritional status and children and adolescents’ dental caries experiences: an overview of systematic reviews. J Appl Oral Sci.

[23] Shivakumar S, Srivastava A, C Shivakumar G (2018). Body mass index and dental caries: a systematic review. Int J Clin Pediatr Dent.

[24] Chen D, Zhi Q, Zhou Y, Tao Y, Wu L, Lin H (2018). Association between dental caries and BMI in children: a systematic review and meta-analysis. Caries Res.

[25] Ministério da Saúde (BR) (2025). Secretaria de Atenção Primária à Saúde. Departamento de Estratégias e Políticas de Saúde Comunitária. SB Brasil 2023: Pesquisa Nacional de Saúde Bucal: relatório final.

[26] Moura RN, Paiva SM, Ramos-Jorge J, Pinto RD, Lara JV, Barbosa MC (2025). Social inequities and dental caries in 5-year-old children: a study with results from SB Brasil 2023. Braz Oral Res.

[27] Ferreira CM, Reis ND, Castro AO, Höfelmann DA, Kodaira K, Silva MT (2021). Prevalence of childhood obesity in Brazil: systematic review and meta-analysis. J Pediatr (Rio J).

[28] Pitts N (2004). "ICDAS": an international system for caries detection and assessment being developed to facilitate caries epidemiology, research and appropriate clinical management. Community Dent Health.

[29] Rodrigues TC (2021). Epidemiologia da má-oclusão no Brasil: revisão dos aspectos etiológico e histórico. Rev Cient Multidiscip Núcl Conhec.

[30] World Health Organization (2000). Obesity: preventing and managing the global epidemic.

[31] Food and Agriculture Organization of the United Nations (2019). The state of food security and nutrition in the world: safeguarding against economic slowdowns and downturns.

[32] Gundersen C, Ziliak JP (2015). Food insecurity and health outcomes. Health Aff (Millwood).

[33] Monteiro CA, Moubarac JC, Cannon G, Ng SW, Popkin B (2013). Ultra-processed products are becoming dominant in the global food system. Obes Rev.

[34] Departamento Intersindical de Estatística e Estudos Socioeconômicos (2024). Custo da cesta aumenta em todas as cidades do Norte e Nordeste.

[35] Instituto Brasileiro de Geografia e Estatística (2020). Pesquisa de orçamentos familiares 2017-2018: análise do consumo alimentar pessoal no Brasil.

[36] Salvato MA, Ferreira PC, Duarte AJ (2010). O impacto da escolaridade sobre a distribuição de renda. Estud Econ.

[37] Black RE, Allen LH, Bhutta ZA, Caulfield LE, Onis M, Ezzati M (2008). Maternal and child undernutrition: global and regional exposures and health consequences. Lancet.

[38] Nowak-Szczepanska N, Gomula A, Ipsen MJ, Koziel S (2016). Different effects of living conditions on the variation in BMI and height in children before the onset of puberty. Eur J Clin Nutr.

[39] Acs G, Lodolini G, Kaminsky S, Cisneros GJ (1992). Effect of nursing caries on body weight in a pediatric population. Pediatr Dent.

[40] Ayhan H, Suskan E, Yildirim S (1996). The effect of nursing or rampant caries on height, body weight and head circumference. J Clin Pediatr Dent.

[41] Alvarez JO, Navia JM (1989). Nutritional status, tooth eruption, and dental caries: a review. Am J Clin Nutr.

[42] Dimaisip-Nabuab J, Duijster D, Benzian H, Heinrich-Weltzien R, Homsavath A, Monse B (2018). Nutritional status, dental caries and tooth eruption in children: a longitudinal study in Cambodia, Indonesia and Lao PDR. BMC Pediatr.

[43] Losso EM, Tavares MC, Silva JY, Urban CA (2009). Severe early childhood caries: an integral approach. J Pediatr (Rio J).

